# Supplementary material for: Single-cell RNA-sequencing analysis of estrogen- and endocrine-disrupting chemical-induced reorganization of mouse mammary gland
Source: Commun Biol. 2019 Nov 5;2:406. doi: 10.1038/s42003-019-0618-9 (PMC6831695; doi:10.1038/s42003-019-0618-9)
Supplement: Supplementary file 1 — Supplementary Information [file 42003_2019_618_MOESM1_ESM.pdf]

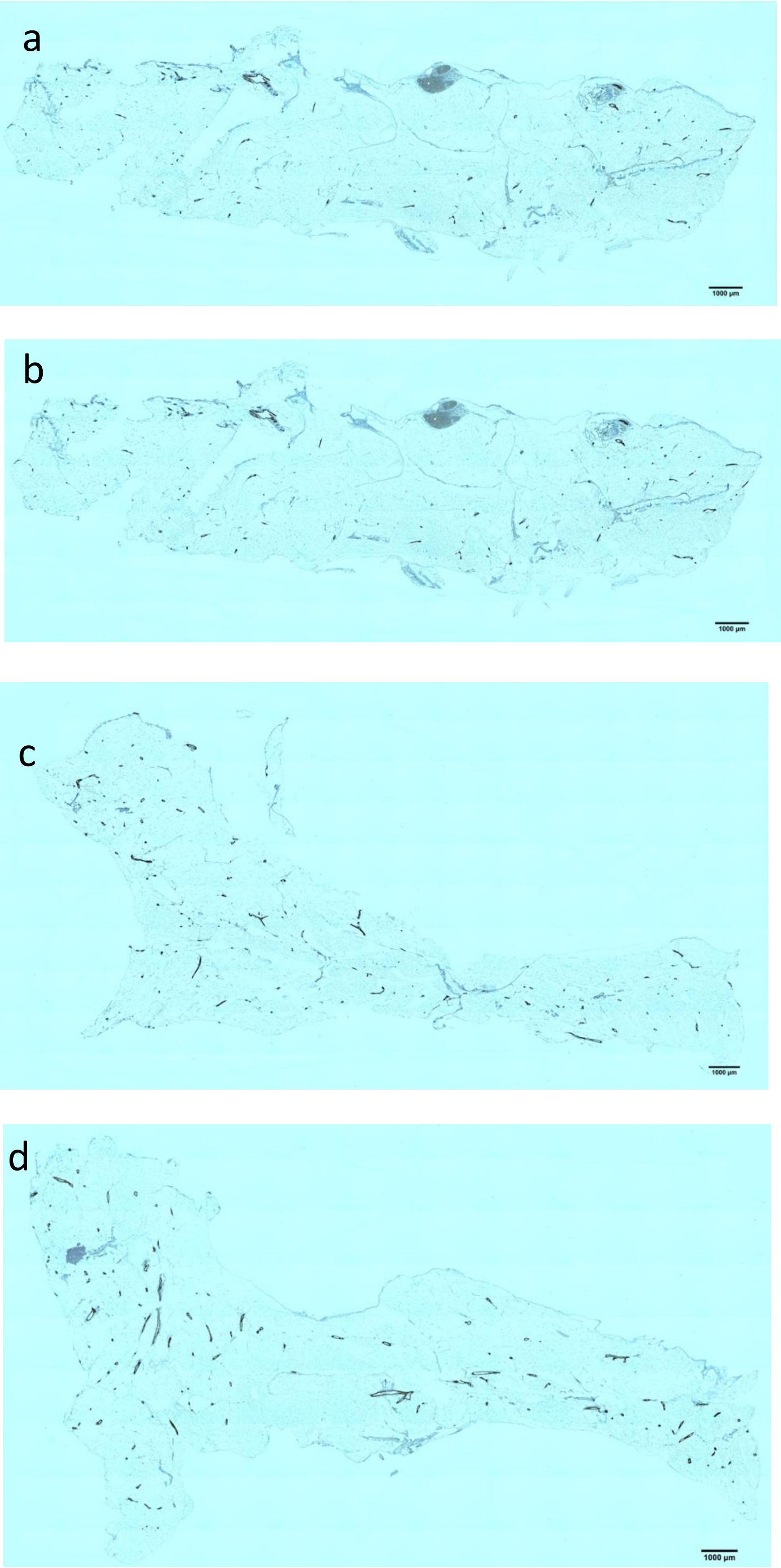

Supplementary Fig. 1. Representative IHC slides stained for KRT18 (1:2000) used for duct quantification. **a**-**d** are whole slide scans of the mammary gland taken from vehicle, PBDE, E2, and E2 + PBDE treated groups respectively. **a** and **b** (vehicle and PBDE) glands contain widely scattered, thin ducts, whereas **c** and **d** glands (E2 and E2 + PBDE) glands have larger ducts. Scale bar = 1000 µm.

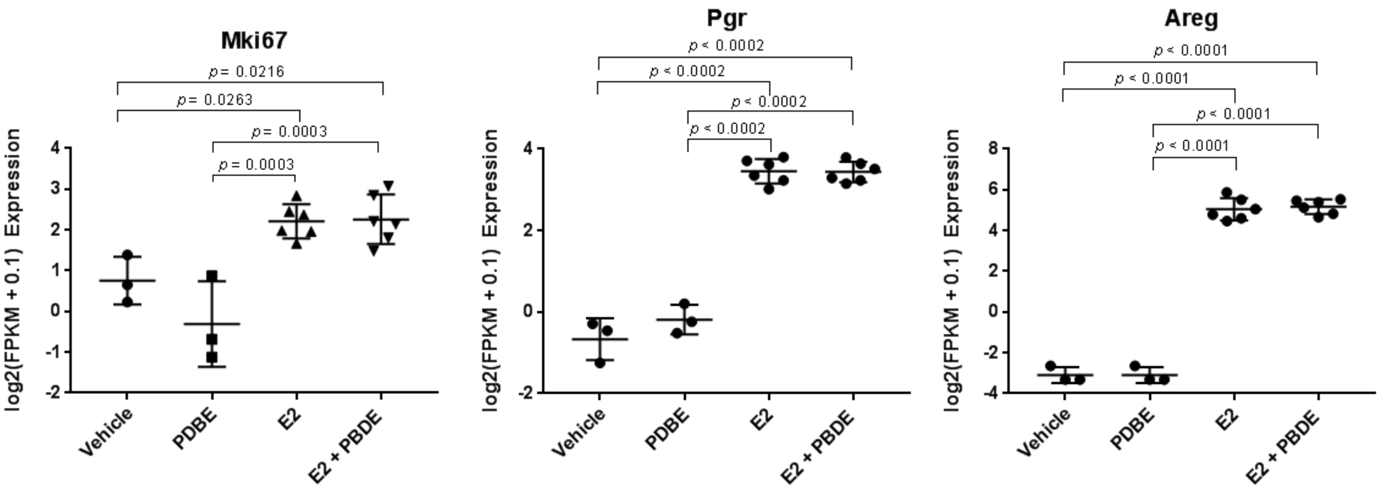

Supplementary Fig. 2. Expression levels of *Mki67*, *Pgr*, and *Areg* from bulk RNAseq data. Expression levels were compared across vehicle treated (n=3), PBDE treated (n=3), E2 treated (n=6), and E2 + PBDE treated (n=6) mice. Tukey's multiple comparisons test was performed.

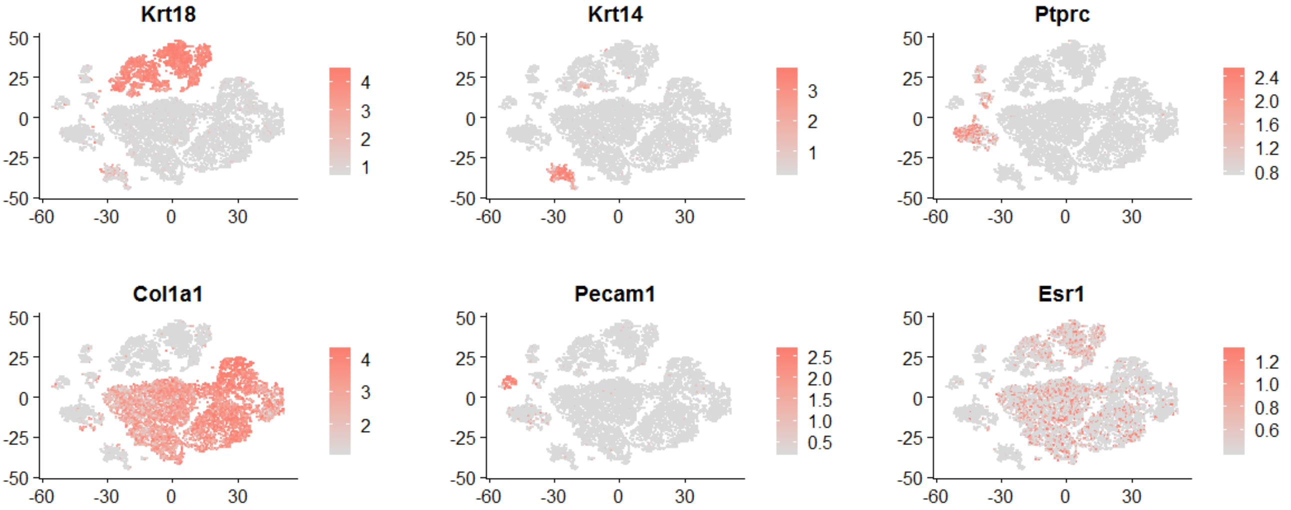

Supplementary Fig. 3. Feature plots of selected cellular markers of different cell types found in literature. *Krt18* (epithelial cells), *Krt14* (basal cells), *Ptprc* (immune cells), *Col1a1* (ECM/fibroblasts), and *Pecam1* (endothelial cells) are used as specific markers to distinguish unique populations among the total 11 clusters. *Esr1* expressing cells were not localized to any specific cluster. These markers identified populations of mammary epithelial cells, endothelial cells, immune cells and fibroblasts.

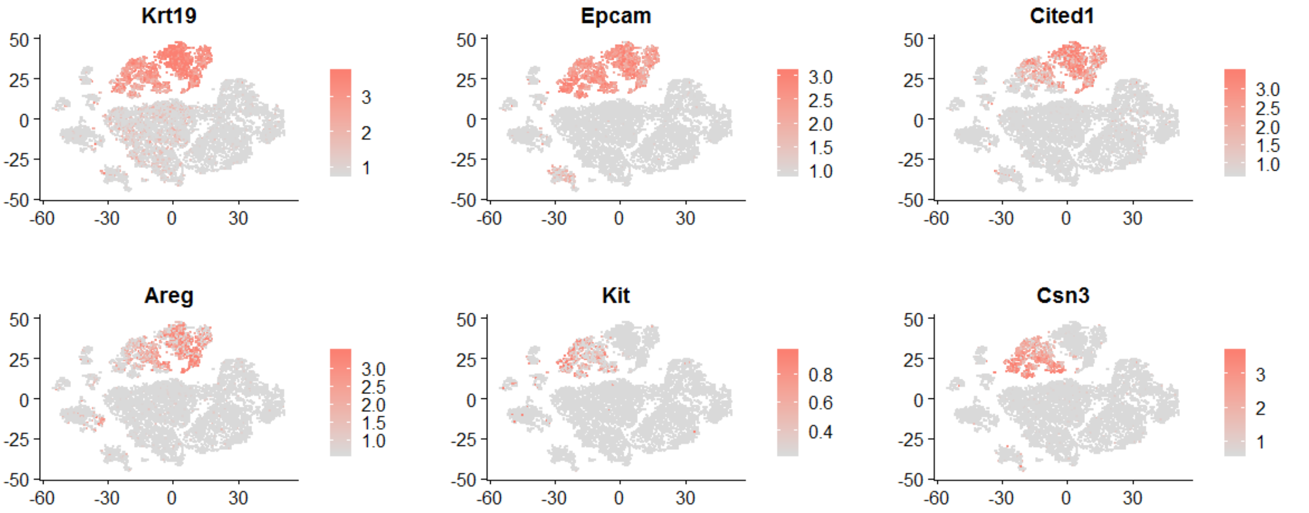

Supplementary Fig. 4. Feature plots of selected luminal epithelial cell markers on the original 11 clusters. *Krt19* and *Epcam* remain ubiquitously expressed in all luminal epithelial cells. More specific markers identified subpopulations of luminal epithelial cells, such as *Cited1* and *Areg* for mature luminal epithelial cells, and *Kit* and *Csn3* for progenitor cells.

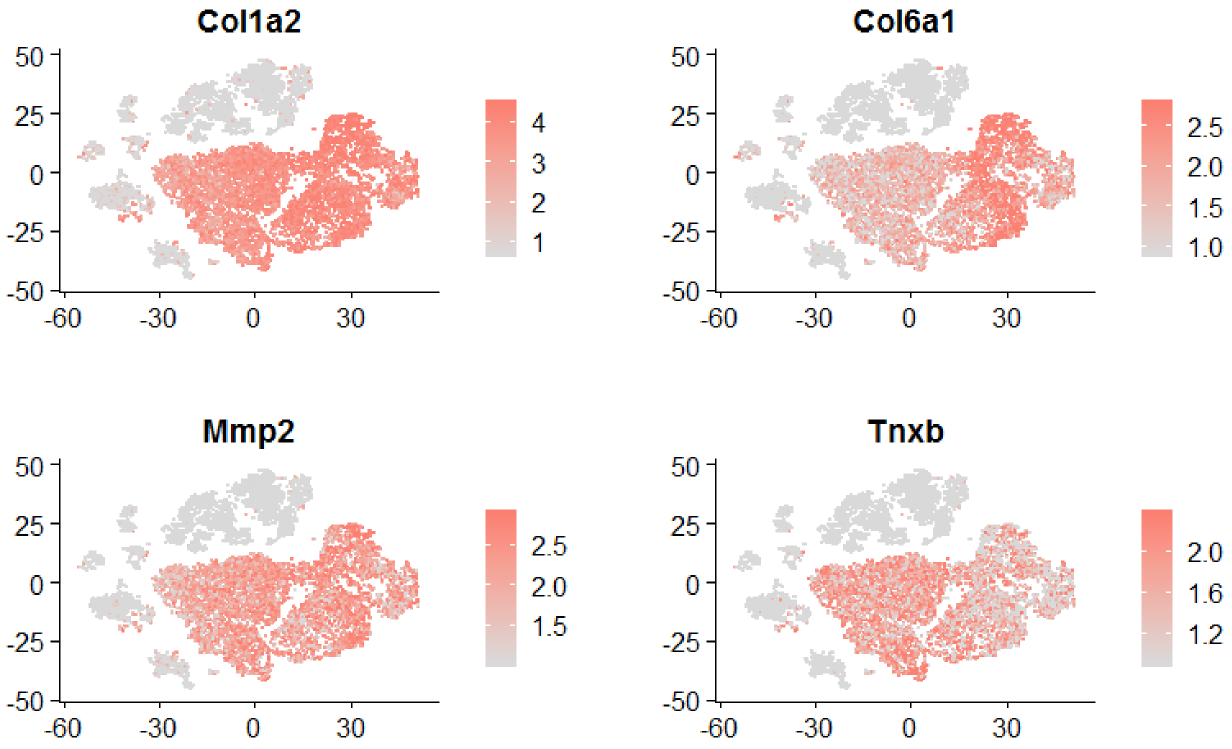

Supplementary Fig. 5. Feature plots of selected fibroblast markers. *Col1a2*, *Col6a1*, *Mmp2*, and *Tnxb* are characteristic stromal fibroblast markers.

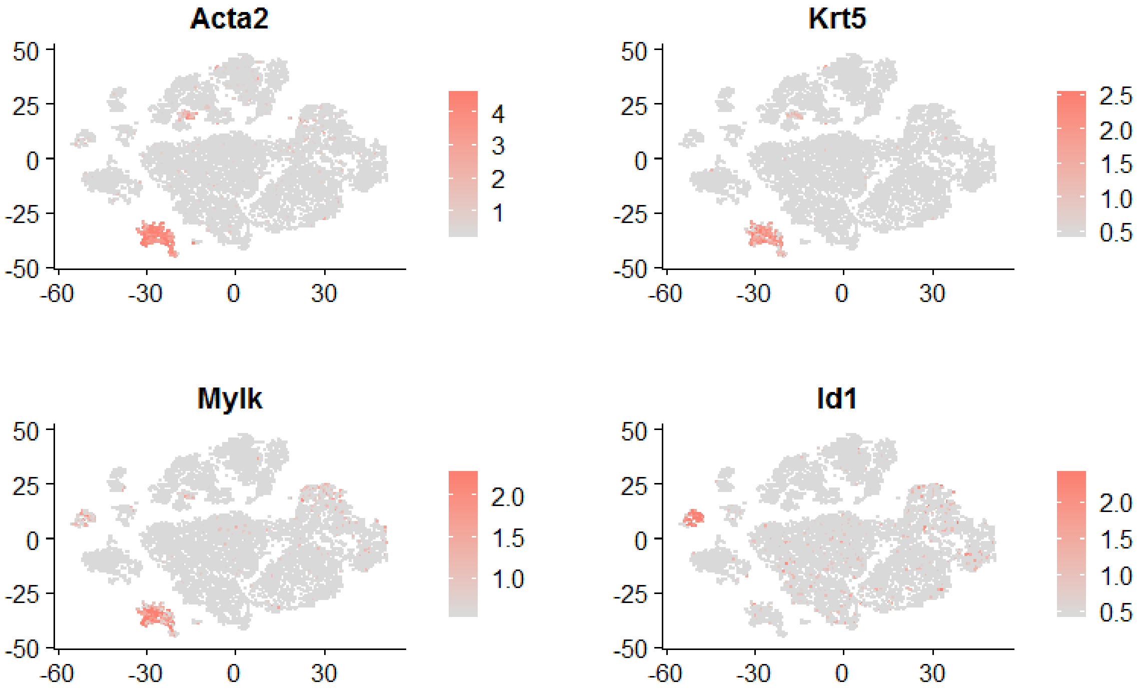

Supplementary Fig. 6. Feature plots of selected basal and endothelial cell markers. *Acta2* is a marker for myoepithelial cells, and both *Krt5* and *Mylk* are smooth cell markers. *Id1* is a marker associated with endothelial cells.

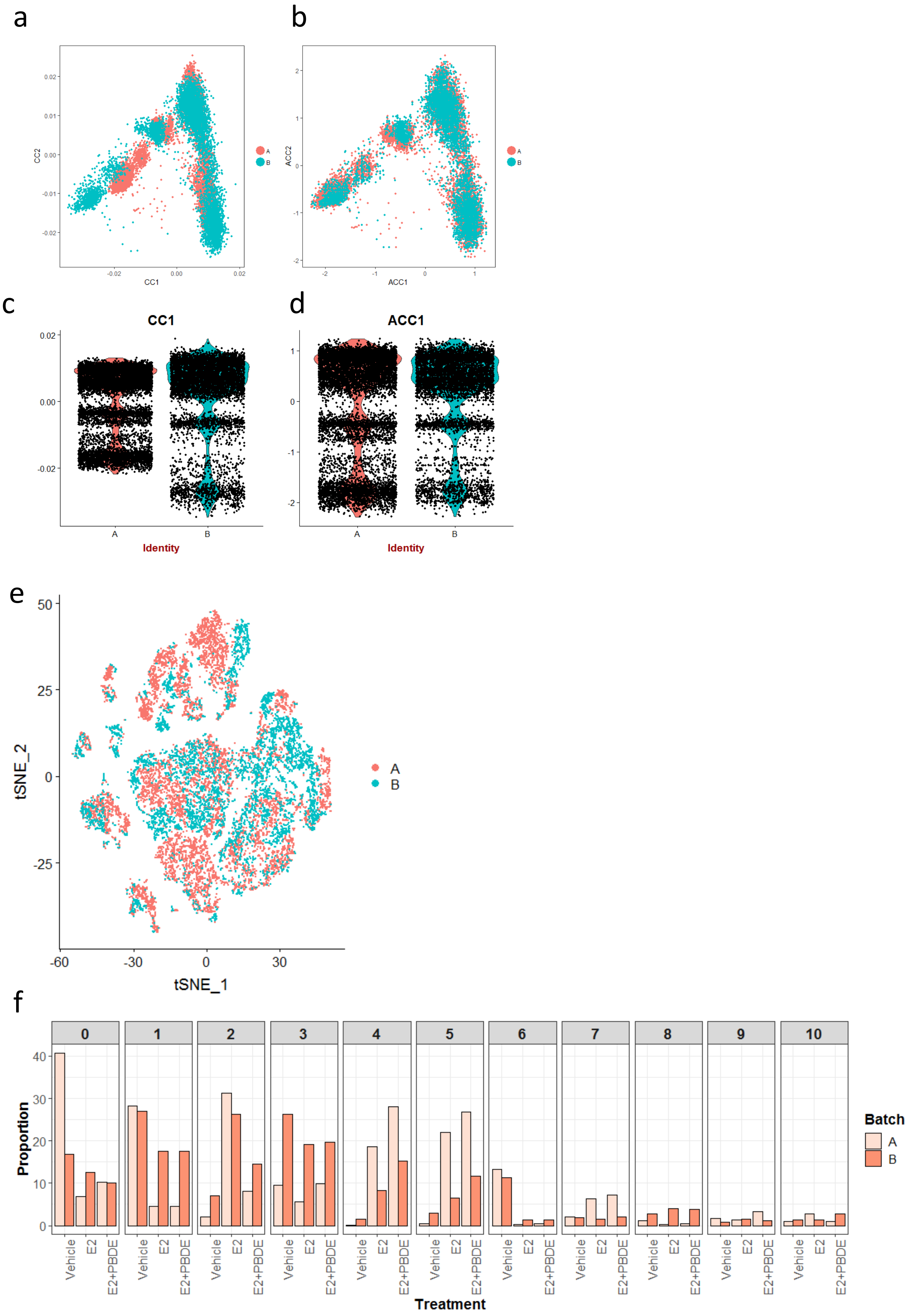
a
b
c
d
e
f
CC1
CC2
ACC1
ACC2
A
B
Identity
tSNE_1
tSNE_2
Proportion
Treatment
Batch
Vehicle
E2
E2+PBDE
0
1
2
3
4
5
6
7
8
9
10

Supplementary Fig. 7. Combination of 2 independent scRNAseq experiments by removal of batch effect between 2 biological replicates. After subspace alignment of canonical correlation, sequencing results from two different batch evenly distributed in ACC spaces. Sample distribution before (a) and after (b) subspace alignment in the first two CC (canonical correlation analysis subspace) and ACC (aligned CC) dimensions, respectively. (c-d): visualization of effective batch removal in violin plots describing sample distribution before (c, CC1 value) and after (d, ACC1 value) subspace alignment. (e) tSNE plot based on dimension reduction and clustering using ACC subspaces, color coded by batches. (f) C0-10 cluster distribution in each batch.

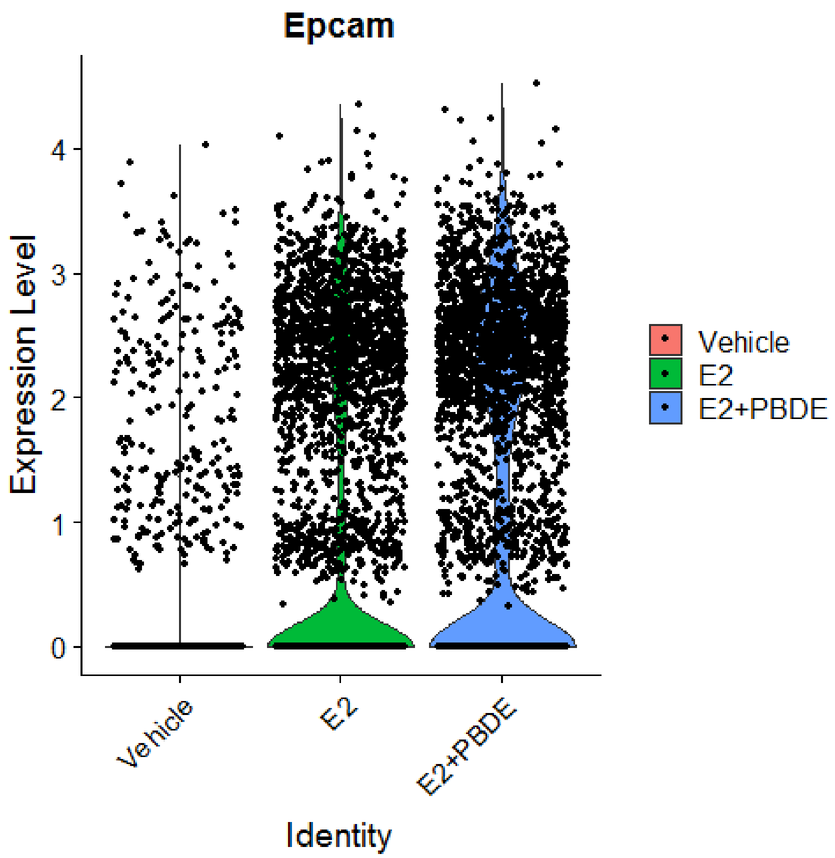

Supplementary Fig. 8. Violin plot of *Epcam* expression in cells from vehicle, E2, and E2 + PBDE treated groups. The number of cells expressing *Epcam* increased after E2 treatment compared to vehicle treatment, consistent with prior whole mount and immunohistochemistry analysis.

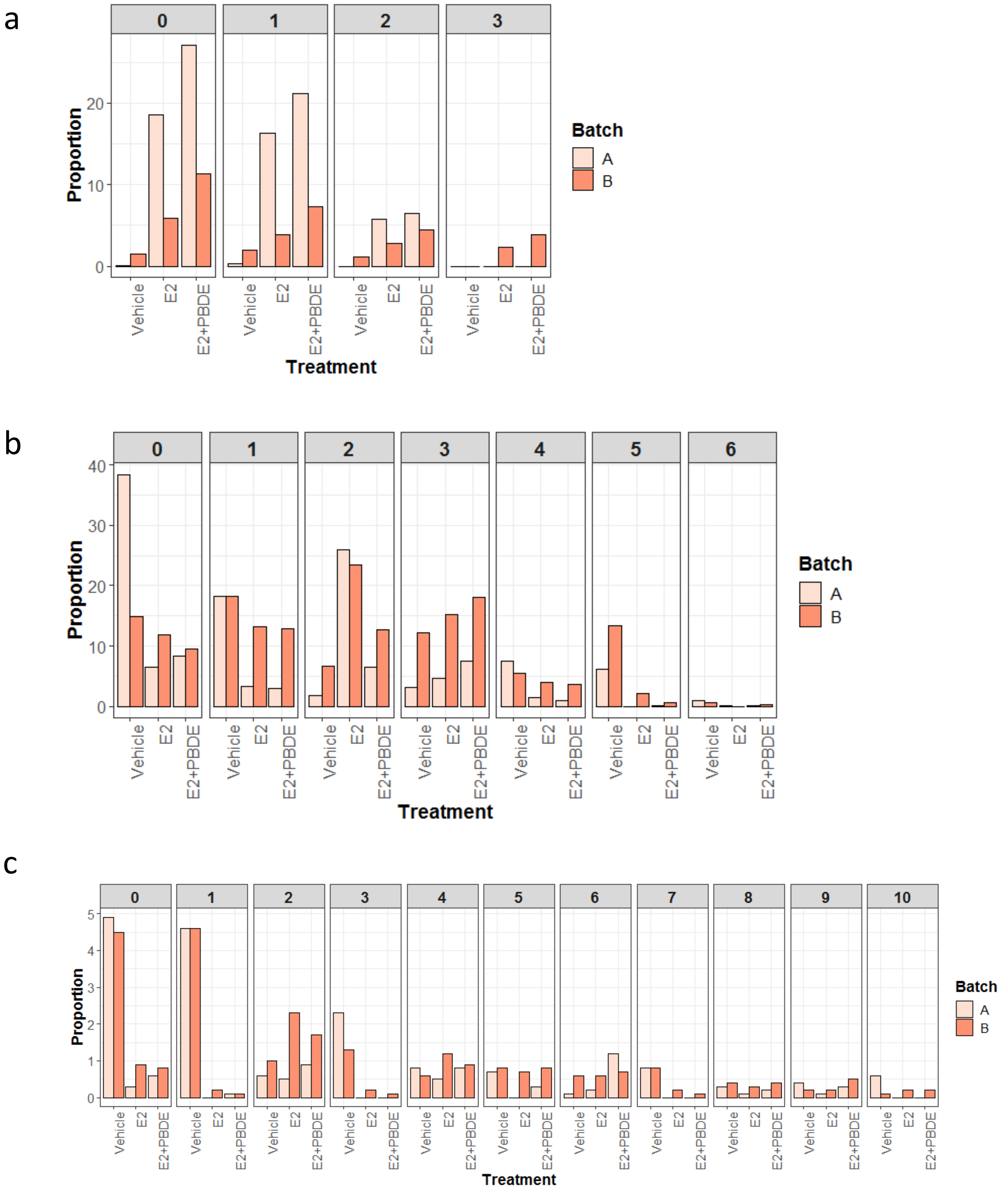

Supplementary Fig. 9. Cluster distribution of two biological replicates (Batch A and B) for (a) luminal cells, (b) fibroblasts, and (c) immune cells. Values are presented as percentage of the total number of cells from each treatment group.

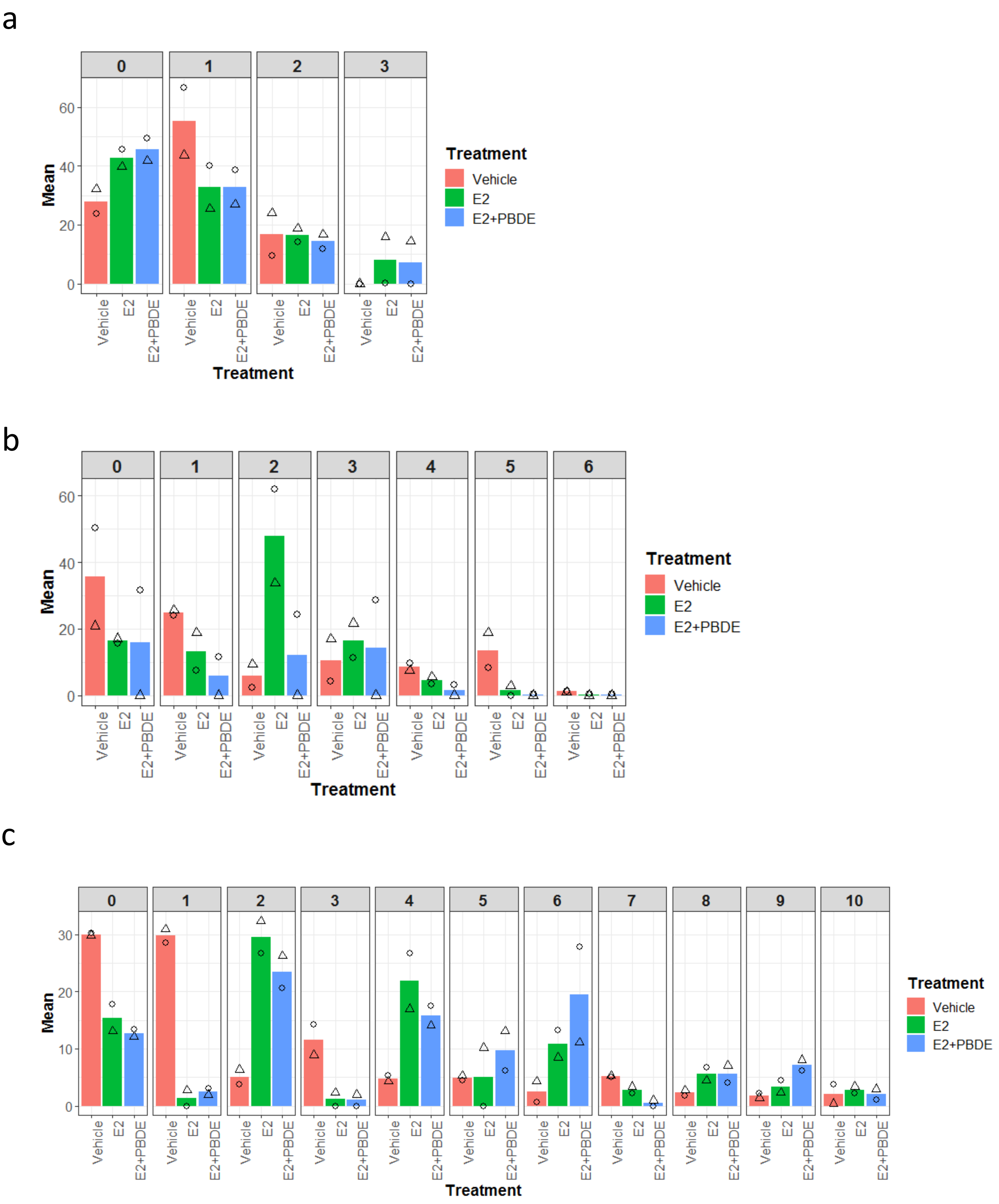

Supplementary Fig. 10. Average cluster distribution of two biological replicates (Batch A and B) for (a) luminal cells, (b) fibroblasts, and (c) immune cells. Values are presented as a percentage of the total number of cells per treatment group, and symbols indicate individual cell distributions from each of the two experimental replicates (△,○).

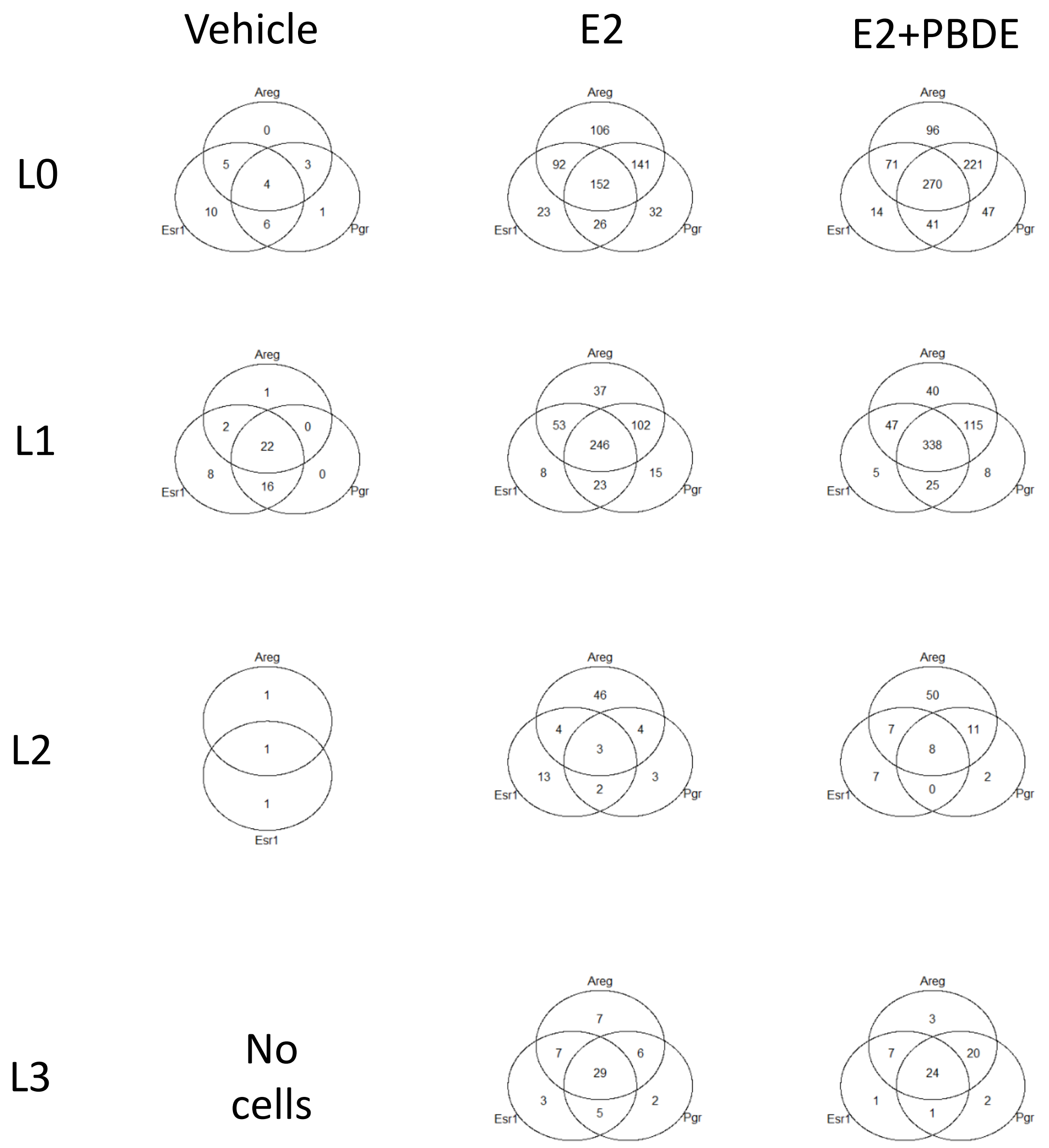

Supplementary Fig. 11. Number of cells in the luminal clusters expressing *Areg, Esr1*, and *Pgr*. Most cells that were *Areg*+ were also *Pgr*+, and were primarily found in the "E2-present" groups.

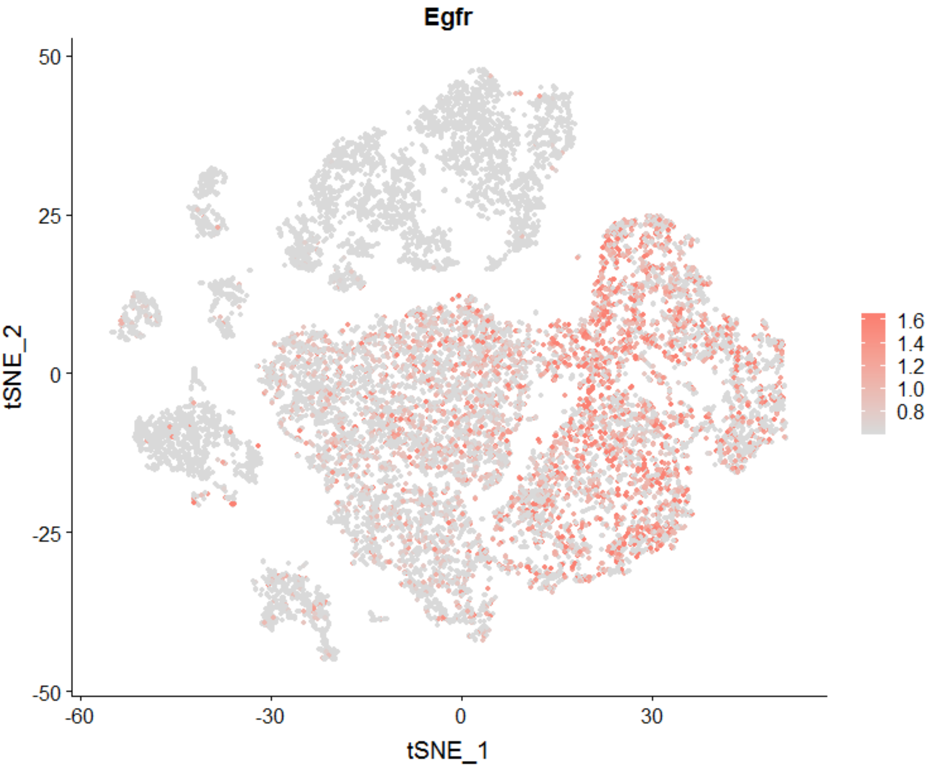

Supplementary Fig. 12. Feature plot of *Egfr* expression on the 11 total clusters. *Egfr* expression was found in the ECM/fibroblast clusters, C0-C3.

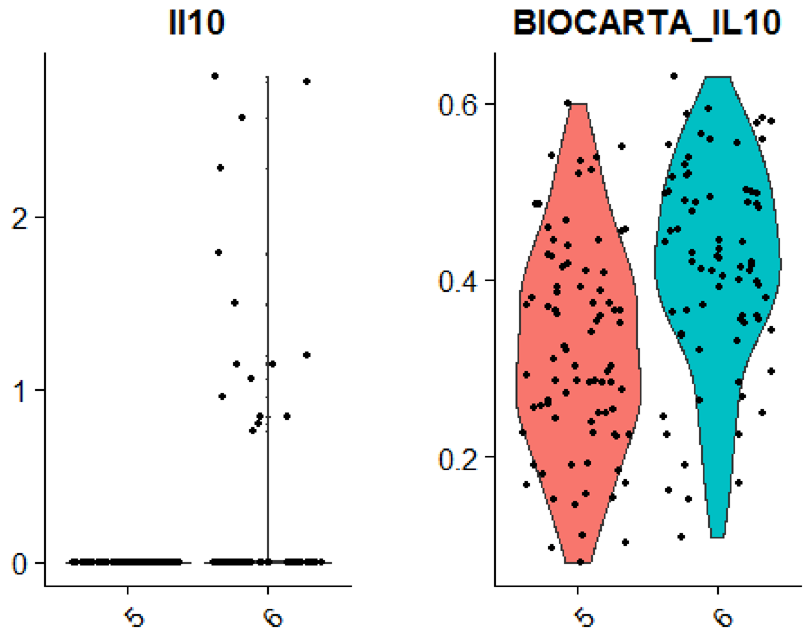

Supplementary Fig. 13. Violin plot of *Il10* expression level and GSVA scores of the BIOCARTA_IL10 gene set for each cell in I5 and I6.

Supplementary Table 1. Number of differentially expressed genes in mouse mammary glands by comparing different treatment groups

Comparison	Up-Regulated	Down-Regulated
E2 vs Vehicle	558	193
PBDE vs Vehicle	0	51
E2 + PBDE vs Vehicle	442	47
E2 + PBDE vs E2	608	5

Supplementary Table 2. Number of cells per cluster

ID	Vehicle	E2	E2+PBDE
C0	2044	486	383
C1	1703	557	374
C2	217	1421	408
C3	895	618	527
C4	36	658	860
C5	72	699	778
C6	775	40	33
C7	122	193	193
C8	99	107	71
C9	89	74	92
C10	69	99	64

Supplementary Table 3. Hormone receptor status of luminal cells by treatment

	ER+/PR-	ER-/PR+	DN	DP
Vehicle	27*	4	29	48
E2	203	305	363	486
E2 + PBDE	159	426	346	707

*Values represent number of cells.
