# Supplementary material for: Single-cell RNA-sequencing analysis of estrogen- and endocrine-disrupting chemical-induced reorganization of mouse mammary gland
Source: Commun Biol. 2019 Nov 5;2:406. doi: 10.1038/s42003-019-0618-9 (PMC6831695; doi:10.1038/s42003-019-0618-9)
Supplement: Supplementary file 2 — Description of Additional Supplementary Items [file 42003_2019_618_MOESM2_ESM.docx]

**Description of additional supplementary items**

Supplementary Data 1 – Top 20 differentially expressed genes per cluster from overall clustering analysis.

Supplementary Data 2 – Top 20 differentially expressed genes per cluster from luminal cell reclustering.

Supplementary Data 3 – Top 20 differentially expressed genes per cluster from fibroblasts reclustering.

Supplementary Data 4 – Top 20 differentially expressed genes per cluster from immune cells reclustering.

Supplementary Data 5 – Data used to generate Figures 1d, 1e, and 4e.
