# Supplementary material for: Single-cell RNA-sequencing analysis of estrogen- and endocrine-disrupting chemical-induced reorganization of mouse mammary gland
Source: Commun Biol. 2019 Nov 5;2:406. doi: 10.1038/s42003-019-0618-9 (PMC6831695; doi:10.1038/s42003-019-0618-9)
Supplement: Supplementary file 8 — Reporting Summary [file 42003_2019_618_MOESM8_ESM.pdf]

nature research

Corresponding author(s): Shiuan Chen

Last updated by author(s): Aug 28, 2019

# Reporting Summary

Nature Research wishes to improve the reproducibility of the work that we publish. This form provides structure for consistency and transparency in reporting. For further information on Nature Research policies, see Authors & Referees and the Editorial Policy Checklist.

## Statistics

For all statistical analyses, confirm that the following items are present in the figure legend, table legend, main text, or Methods section.

| n/a | Confirmed | |
|---|---|---|
| ☐ | ☒ | The exact sample size (*n*) for each experimental group/condition, given as a discrete number and unit of measurement |
| ☐ | ☒ | A statement on whether measurements were taken from distinct samples or whether the same sample was measured repeatedly |
| ☐ | ☒ | The statistical test(s) used AND whether they are one- or two-sided<br>*Only common tests should be described solely by name; describe more complex techniques in the Methods section.* |
| ☐ | ☒ | A description of all covariates tested |
| ☐ | ☒ | A description of any assumptions or corrections, such as tests of normality and adjustment for multiple comparisons |
| ☐ | ☒ | A full description of the statistical parameters including central tendency (e.g. means) or other basic estimates (e.g. regression coefficient) AND variation (e.g. standard deviation) or associated estimates of uncertainty (e.g. confidence intervals) |
| ☐ | ☒ | For null hypothesis testing, the test statistic (e.g. *F*, *t*, *r*) with confidence intervals, effect sizes, degrees of freedom and *P* value noted<br>*Give P values as exact values whenever suitable.* |
| ☒ | ☐ | For Bayesian analysis, information on the choice of priors and Markov chain Monte Carlo settings |
| ☒ | ☐ | For hierarchical and complex designs, identification of the appropriate level for tests and full reporting of outcomes |
| ☒ | ☐ | Estimates of effect sizes (e.g. Cohen's *d*, Pearson's *r*), indicating how they were calculated |

*Our web collection on statistics for biologists contains articles on many of the points above.*

## Software and code

Policy information about availability of computer code

| | |
|---|---|
| Data collection | The following software was used for data collection: Hiseq 2500 system RTA v1.18.64 and 10x Genomics Chromium software Cell Ranger v1.3.1 for Single cell sequencing data. |
| Data analysis | scRNA-seq:Seurat 2.3.2, R v3.5.0, GSEA v3.0 |

For manuscripts utilizing custom algorithms or software that are central to the research but not yet described in published literature, software must be made available to editors/reviewers. We strongly encourage code deposition in a community repository (e.g. GitHub). See the Nature Research guidelines for submitting code & software for further information.

## Data

Policy information about availability of data

All manuscripts must include a data availability statement. This statement should provide the following information, where applicable:

- Accession codes, unique identifiers, or web links for publicly available datasets
- A list of figures that have associated raw data
- A description of any restrictions on data availability

All raw and processed scRNA-seq data have been uploaded to the Array Express database GEO with the accession number GSE125272. A secure token was created to allow reviewer access: czunmqkmhvkldon.

# Field-specific reporting

Please select the one below that is the best fit for your research. If you are not sure, read the appropriate sections before making your selection.

☒ Life sciences ☐ Behavioural & social sciences ☐ Ecological, evolutionary & environmental sciences

For a reference copy of the document with all sections, see nature.com/documents/nr-reporting-summary-flat.pdf

# Life sciences study design

All studies must disclose on these points even when the disclosure is negative.

| | |
|---|---|
| Sample size | Sample size was determined based on the other studies (i.e. Aupperlee et al., Endocrinology, 2014) which they used similar phenotypic analysis (i.e. terminal end buds count using mammary gland whole mount staining). |
| Data exclusions | No data exclusions |
| Replication | Duplicate single-cell preparations from two independent experiments were collected and processed for scRNAseq |
| Randomization | Randomization was performed before surgeries. Moreover, OVX mice were randomized into four groups (vehicle, E2, PBDE, or E2+PBDE) for treatment. |
| Blinding | scRNAseq results was generated by individual who did not know the treatment information of each group (blinding) . |

# Reporting for specific materials, systems and methods

We require information from authors about some types of materials, experimental systems and methods used in many studies. Here, indicate whether each material, system or method listed is relevant to your study. If you are not sure if a list item applies to your research, read the appropriate section before selecting a response.

## Materials & experimental systems

| n/a | Involved in the study | |
|---|---|---|
| ☐ | ☒ | Antibodies |
| ☒ | ☐ | Eukaryotic cell lines |
| ☒ | ☐ | Palaeontology |
| ☐ | ☒ | Animals and other organisms |
| ☒ | ☐ | Human research participants |
| ☒ | ☐ | Clinical data |

## Methods

| n/a | Involved in the study | |
|---|---|---|
| ☒ | ☐ | ChIP-seq |
| ☒ | ☐ | Flow cytometry |
| ☒ | ☐ | MRI-based neuroimaging |

## Antibodies

| | |
|---|---|
| Antibodies used | Anti-Estrogen Receptor α rabbit Antibody (Millipore Sigma, 06-935); Anti-Progesterone Receptor rabbit Antibody (Abcam, ab131486); Ki-67 (DB35) Rabbit mAb (Cell Signaling Technology, 12202S); Recombinant Anti-Cytokeratin 18 Antibody (Abcam, ab181597), Mach 2 Rabbit HRP-Polymer (Biocare Medical, RHRP520L) |
| Validation | ER and PR antibodies were used in Catanese et al's paper (Endocrinology, 2017) for mouse tissues and validated using B6C3F1 Mouse Uterus Tissue (Diestrus) as positive control. Ki67 antibody was validated using mouse intestine as positive control. Krt18 antibody was validated using mouse liver as positive control. |

## Animals and other organisms

Policy information about studies involving animals; ARRIVE guidelines recommended for reporting animal research

| | |
|---|---|
| Laboratory animals | Female BALB/cj, 9 weeks old |
| Wild animals | N/A |
| Field-collected samples | N/A |
| Ethics oversight | All animal research procedures used in this study were approved by the Institutional Animal Care and Use Committee (IACUC) at City of Hope. |

Note that full information on the approval of the study protocol must also be provided in the manuscript.
